# *Aspergillus flavus* YRB2 from *Thymelaea hirsuta* (L.) Endl., a non-aflatoxigenic endophyte with ability to overexpress defense-related genes against Fusarium root rot of maize

**DOI:** 10.1186/s12866-022-02651-6

**Published:** 2022-09-30

**Authors:** Younes M. Rashad, Sara A. Abdalla, Ahmed S. Shehata

**Affiliations:** 1 Plant Protection and Biomolecular Diagnosis Department, Arid Lands Cultivation Research Institute, City of Scientific Research and Technological Applications (SRTA-City), New Borg El-Arab City, 21934 Egypt; 2 Environment and Natural Materials Research Institute (ENMRI), City of Scientific Research and Technological Applications (SRTA-City), New Borg El-Arab City, 21934 Egypt

**Keywords:** Antifungal, Biocontrol, *Fusarium solani*, *JERF3*, *Zea mays*, Mitnan

## Abstract

Fusarium root rot, caused by *Fusarium solani* (Mart.) Sacc., represents one of the most damaging diseases of maize affecting plant growth and yield. In this study, the antagonistic potential of a non-aflatoxigenic endophytic *Aspergillus flavus* YRB2, isolated from *Thymelaea hirsuta* (L.) Endl., was tested against *F. solani* in vitro. In addition, its biocontrol activity against Fusarium root rot of maize was evaluated under greenhouse conditions. Its impacts on plant molecular, pathological, physiological, and growth levels were also studied. Results obtained revealed a potent antagonistic behavior for *A. flavus* YRB2 against *F. solani* in vitro, recording 80% growth inhibition. Seventeen secondary metabolites were detected in the n-hexane extract of *A. flavus* YRB2 filtered culture broth using GC-MS analysis. Among them, various antifungal secondary metabolites were produced, namely palmitic acid, α-linolenic acid, stearic acid, 2, 4-di-tert-butylphenol, diisobutyl phthalate, and heneicosane. In contrast, HPLC analysis showed that no aflatoxins (B1, B2, G1, and G2) were detected. Under greenhouse conditions, colonization of maize plants with *A. flavus* YRB2 exhibited a potential biocontrol activity against Fusarium root rot, recording 73.4% reduction in the disease severity. Triggering of transcriptional expression level of the defense-related genes *JERF3* (7.2-fold), *CHI II* (8-fold), and *POD* (9.1-fold) was reported, indicating the inducing effect on the plant immunity. In addition, an increment in the antioxidant enzymes POD and PPO, and the total phenolic content in maize roots was also observed in response to this treatment. Moreover, a growth-promoting effect was also observed for colonization of maize plants with *A. flavus* YRB2. Based on the obtained data, we can conclude that *A. flavus* YRB2 may represent a promising biocontrol and growth-promoting agent for maize plants against Fusarium root rot. Nevertheless, field evaluation is highly requested before the use recommendation.

## Introduction

*Zea mays* L., commonly known as maize, is an annual crop that belongs to family Poaceae. In 2020, maize ranked first as the most produced cereal worldwide with a total production around 1.2 billion tons from a total harvested area of 202 million ha [1]. The high economic value of maize is due to its multiple uses such as human food, animal feed, and a raw ingredient in many industries such as starch, oil, alcohol, and fuel production. However, its processing and consumption vary greatly from one country to another [2]. Maize has a high nutritional value containing starch (60–70%), protein (8–10%), oil (4–6%), in addition to vitamins, essential minerals and dietary fibers [3]. This makes maize a staple food and a good source of energy for many people worldwide. However, maize crop is exposed to infection with around 112 fungal, bacterial, viral, and nematode diseases that cause high damages and yield loss up to 80%, such as rots, blights, downy mildew, wilt, rusts, and smuts [4].

Fusarium root rot, caused by *Fusarium solani* (Mart.) Sacc., is one of the most damaging diseases of maize. The pathogen overwinters as mycelium or spores in soil and plant residues. The diseased seedlings show brown to black discoloration, mainly in the hypocotyl region, rotted roots, which may die in severe infection [5]. However, the disease diagnosis is relatively complicated due to the ability of multiple *Fusarium* spp. to cause root rot in maize, including *F. solani* [6]. In addition, different *Rhizoctonia* spp. and *Pythium* spp. can also cause maize root rot [7, 8]. Due to concerns about their health and environmental risks, use of chemical fungicides to control plant diseases is undesirable [9]. Several attempts have been made to develop safe alternatives. Biological control using different fungal and bacterial isolates represents an effective and safe option in this regard [10].

Endophytic fungi can be defined as the fungi that inhabit plant tissues during at least a part of their life cycle without causing any disease symptoms or damage [11]. Fungal endophytes have received a wide interest in the recent years owing to their unrevealed and distinctive properties, and unique bioactive secondary metabolites that support their mutualistic association with the hosts and induce host resistance to different biotic and abiotic stresses [12]. Improving plant growth and triggering its defense responses to different invading pathogens by endophytic fungi have been widely investigated [13]. Tseng et al. [14] reported a new endophytic *Trichoderma* strain, isolated from leaves of *Leucas aspera*, which can colonize roots of *Arabidopsis thaliana* and *Nicotiana attenuate* promoting their growth, and protecting their roots against infection with *Alternaria brassicicola.* In addition, it showed an antifungal effect of mycelial growth of *A. brassicicola.* In another study, Wei et al. [15] evaluated an endophytic isolate (*F. solani* CEF559) against *Verticillium dahliae*, the causal agent of Verticillium wilt of cotton. This endophyte showed a potent antagonistic activity against *V. dahliae* resulting in a 75% growth suppression, and a full inhibition in the conidial production. Moreover, it reduced the disease severity in cotton plants by 56.3% when applied under field conditions. Biocontrol activity of *F. solani* CEF559 was discussed in the light of their resistance-inducing effect via overexpression of multiple pathogenesis-related (PR) genes and lignin biosynthesis-related genes, as well as their direct antifungal activity against the pathogen. Recognition of mechanisms involved in the biocontrol behavior of the endophyte is crucial to achieve effective and sustainable plant disease protection. In this concern, various mode of actions have been discussed including competition for space and/or nutrients, antibiosis by production of antifungal volatile and/or non-volatile metabolites, and/or direct mycoparasitism [16]. All or some of these mechanisms may contribute in the antagonistic behavior of the endophyte resulting in inhibition of mycelial growth, sporulation, and/or spore germination of the fungal pathogen [17]. In addition, induction of host immunity to the invading pathogen via eliciting different signaling pathways is another indirect mechanism that may be involved in the biocontrol activity of endophytes [18]. Induction of plant resistance leads to activation of multiple hypersensitivity reactions in the host including lignin deposition, accumulation of fungitoxic phenolic compounds, stimulation the host plant to produce antifungal metabolites (phytoalexins), triggering different PR proteins such as 1, 3-glucanases and chitinases, and/or overexpression of various defense-related genes [19]. Among the up-regulated genes, jasmonate and ethylene responsive factor 3 (*JERF3*), which regulates a set of defense-responsive genes via jasmonate/ethylene signaling pathways, has been reported to be activated by colonization of beneficial endophytes [20, 21]. Chitinase (*CHI*) gene, which encodes the lytic enzyme chitinase, is an antifungal defensive gene against different pathogenic fungi. It catalyzes degradation of chitin, the main structural unit of the cell wall in fungi [22]. Up-regulation of peroxidase gene (*POD*), which encodes the antioxidant peroxidase enzyme, is one of the reported defense responses against many invading fungal pathogens scavenging the resultant reactive oxygen species (ROS) [23]. This study aimed to 1) investigate the antagonistic activity of the endophytic *Aspergillus flavus* YRB2 against *F. solani* in vitro, 2) identify its produced antifungal secondary metabolites, 3) test its aflatoxigenic ability, 4) evaluate its biocontrol activity against Fusarium root rot of maize under greenhouse conditions, 5) investigate its effect on the transcriptional level of the defense-related genes *JERF3*, *PR 1* and *CHI II* in maize, and 6) study its impact on the total phenolic content, activity of the antioxidant enzymes polyphenoloxidase (PPO) and peroxidase (POD), as well as the growth parameters of maize.

## Materials and methods

### Maize cultivar and fungal isolates

Maize grains of Giza 128 cultivar, kindly provided by Central Administration for Seed Certification, Egypt, were used in the greenhouse experiment. A potent isolate of *F. solani*, originally isolated from rotted maize roots, was obtained from Plant Pathology Research Institute, Agricultural Research Center (ARC), Giza, Egypt. The fungal pathogen was kept on potato dextrose agar (PDA, Difco, Detroit, MI, USA) slants at 4 °C until use. The endophytic strain was isolated from roots and stem of *Thymelaea hirsuta* (L.) Endl. (Mitnan), a wild plant grown in an arid region near New Borg El-Arab City, Egypt. This plant was identified by Prof. I.M. Mashaly, and deposited in the Herbarium of Botany Department, Faculty of Science, Mansoura University, Egypt (D.N.: 030–30-83,201).

To isolate the endophytic strain, plant sections surface sterilized in sodium hypochlorite solution (5%), were plated on PDA medium and incubated at 28 °C for 3 days. The endophyte was purified using the single spore technique, designated YRB2, and morphologically identified according to its colony features, and microscopical characteristics according to Raper and Fennell [24], and Pitt and Hocking [25]. The endophytic strain was identified as *A. flavus* YRB2. The identity of the isolated fungus was molecularly confirmed using internal transcribed spacer (ITS) region of DNA. For molecular identification, DNA was extracted using QiAamp DNA Mini Kit (Qiagen, Hilden, Germany). To amplify ITS region (600 bp) of DNA, the universal fungi primers ITS1 (5’TCCGTAGGTGAACCTTGCGG3’) and ITS4 (5’TCCTCCGCTTATTGATATGC3’) were used following the method adopted by White et al. [26]. The PCR product was sequenced (Macrogene Co., Seoul, Korea). The nucleotide sequence was aligned and compared with the GenBank database via the NCBI search tool BLAST. The phylogenetic tree of the obtained sequence and the closest BLAST sequences from the GenBank database was constructed based by the maximum likelihood method, using MEGA X software version 10.2.4 [27]. For inocula preparation, each fungus was grown on PDA plates at 28 °C for 7 days. Conidia of each fungus were harvested using sterile water and a conidial suspension was prepared and adjusted at 3 × 10^5^ conidia mL^− 1^.

### Antagonism assay of *A. flavus* YRB2 against *F. solani* in vitro

Dual culture technique was used to assess the antagonistic behavior of the endophytic *A. flavus* YRB2 against *F. solani.* PDA plate was inoculated with a mycelial disc (6 mm diameter), taken from a 7 days-old-culture of *A. flavus* YRB2, 1 cm from the plate edge. One centimeter from the opposite edge of the same plate, a mycelial disc (6 mm diameter), taken from 7 days-old-culture of *F. solani*, was inoculated. PDA plates singly inoculated with a 6 mm-diameter-disc of either *A. flavus* YRB2 or *F. solani* were used as control treatments. All treatments were applied in triplicate. All plates were incubated at 28 ± 2 °C and the inward mycelial growth was measured after 4 and 8 days. Inhibition in the fungal growth was calculated using the following equation:

$$\mathrm{Growth}\ \mathrm{Inhibition}\ \left(\%\right)=\frac{\mathrm{G}1-\mathrm{G}2\ }{\mathrm{G}1}\times 100$$

Where G1 = linear growth in the control plate, and G2 = linear growth in the dual culture plate.

### Assessment of the antifungal activity of extracellular metabolites of *A. flavus* YRB2

Filtrate (100 mL) of a 7 days-old-culture of *A. flavus* YRB2 grown on PD broth (Difco, Detroit, MI, USA) was centrifuged (Centurion Scientific K241R, Chichester, UK) at 6000 rpm for 20 min at 4 °C. The supernatant was used for successive extraction with two solvents of different polarities (n-hexane and ethyl acetate, 250 ml). The respective organic phases were collected, dried with sodium sulfate anhydrous, and concentrated by evaporating the excess solvent using a rotary evaporator (IKA RV3, Staufen, Germany). Agar well diffusion method was used to test the antifungal activity of both extracts. Three millimeters from the edge of a PDA plate, a 6 mm-diameter-well was made using a sterile cork borer. For each extract, 100 μL was singly added to a respective well. A mycelial disc (6 mm diameter), taken from 7 days-old-culture of *F. solani*, was inoculated 0.5 cm opposite to the well. For control treatments, 100 μL of each solvent were singly added to a well instead of the extract. Five replicates were used for each treatment. Then, the plates were incubated at 28 ± 2 °C for 7 days. Antifungal activity was determined by measuring the inhibition in the mycelial growth compared to the control.

### Gas chromatography/mass spectrometry (GC/MS) analysis

Filtrate of *A. flavus* YRB2 grown on PD broth and incubated at 28 ± 2 °C for 7 days was extracted using n-hexane. Chemical constituents of the n-hexane extract were identified using a GCMS-QP2010 system (Shimadzu, Japan) equipped with a mass selective detector (MS) at Central Laboratory of Egypt-Japan University of Science and Technology (E-JUST), Alexandria, Egypt. The analysis conditions were as follows: detector mass spectrometer voltage 75 eV at max temperature 250 °C. The DB-5HT capillary column (15 m × 0.32 mm × 0.1 μm) was used with helium as a carrier gas at 2.6 mL min^− 1^. The oven temperature was adjusted at 50 °C for 1 min, increased to 180 °C with a rate of 15 °C min^− 1^, held for 1 min at this temperature, increased again to 230 °C with a rate of 7 °C min^− 1^, held for 2 min, and then increased to 250 °C at 10 °C min^− 1^. The injected volume was 2 μL. To identify the components, their retention time and mass spectra were compared with those in the National Institute of Standards and Technology (NIST 11) Spectral Library (Gaithersburg, MD, USA).

### Detection of aflatoxin production using high-performance liquid chromatography (HPLC)

Filtrate of *A. flavus* YRB2 grown on PD broth and incubated at 28 ± 2 °C for 12 days was used to investigate aflatoxin production. Production of aflatoxins B1, B2, G1, and G2 was detected using the HPLC system; Agilent 1100 Series quaternary pump, Agilent 1100 Series autosampler, Agilent 1100 Series MWD multiple-wavelength detector, and HPLC 2D Chemstation software (Agilent Technologies, Santa Clara, CA, USA). The chromatographic separation was performed with a reversed-phase column (Extend-C18, column, 4.6 mm × 250 mm, 5 μm particle size, Agilent Co.). The culture filtrate was analyzed isocratically using 50:40:10 (v/v/v) water/methanol/acetonitrile mixture as the mobile phase. The column temperature adjusted at 35 °C at flow rate of 1 mL min^− 1^ to achieve the optimum resolution of the aflatoxins. The injected volume was maintained at 20 μL for both the sample and standard solutions. The aflatoxins mixture standard solution (B1, B2, G1, and G2) was used for the aflatoxins detection.

### Greenhouse experiment

Maize grains, surface sterilized in 5% sodium hypochlorite solution, were sown in pots (20 cm diameter) filled with sterilized soil at 5 grains per pot. Endophytic *A. flavus* YRB2 was applied by soaking maize grains in its conidial suspension supplemented with 3% gum arabic for 3 h before the sowing. In addition, another dose (5 mL/seedling) of the conidial suspension was applied as a soil drench 21 days post sowing. Maize grains soaked in sterilized water (with 3% gum arabic) were used for the control treatment. Soil infestation was achieved by mixing the conidial suspension of *F. solani* with the upper layer of the soil at 2% (v/v), 14 days post grains sowing. Five pots of each treatment were used. All pots were arranged in a complete randomized design, regularly watered, and kept under greenhouse conditions at 31/25 °C day/night, 70% humidity. The applied treatments were as follows: C: non-colonized with *A. flavus* YRB2 and uninfected with *F. solani*, P: noncolonized with *A. flavus* YRB2 and infected with *F. solani*, E: uninfected with *F. solani* and colonized with *A. flavus* YRB2, and P + E: infected with *F. solani* and colonized with *A. flavus* YRB2. To confirm its colonization in maize plants, endophytic *A. flavus* YRB2 was re-isolated from tissues of the inoculated maize plants (roots, stem, and leaves). For re-isolation of the endophyte, 10 maize grains were surface sterilized using sodium hypochlorite solution, soaked in a spore suspension of *A. flavus* YRB2 (10^6^ spore mL-1), and planted in a twice sterilized soil. After 30 days, 5 maize seedlings were uprooted, washed with tap water, cut into small pieces (1–2 mm), and surface sterilized using sodium hypochlorite solution. The roots, stem, leaves pieces were then arranged on PDA plates at 5 pieces per plate, and incubated at 28 °C for 7 days. The grown fungus was re-isolated and morphologically re-identified under light microscope.

#### Expression profiling of the defense-related genes

Three days post infection (dpi), samples of maize roots from each treatment were collected for molecular study. mRNA extraction of the samples was carried out using RNeasy Mini Kit (Qiagen, Hilden, Germany). The obtained mRNA was quantified by using a NanoDrop 1000 spectrophotometer (Thermo Fisher Scientific Inc., Wilmington, DE, USA), then stored at − 20 °C. For cDNA synthesis, a reaction mixture with a total volume of 20 μL was used containing mRNA (30 ng, 3 μL), dNTPs (10 mM, 2.5 μL), reaction buffer (2.5 μL), primer (5 pmol μL^− 1^, 5 μL), reverse transcriptase enzyme (New England Biolabs, Germany) (0.2 μL), and RNase free water (6.8 μL). The reaction was carried out using a SureCycler 8800 thermocycler (Agilent, Santa Clara, CA, USA) at 42 °C for 1 h, and at 70 °C for 10 min then the product was stored at − 80 °C.

The quantitative real-time PCR (qRT-PCR) performed using a Rotor-Gene-6000-system (Qiagene, USA). A reaction mixture with a total volume of 20 μL was used containing cDNA (3 μL), SYBR Green Master Mix (Bioline, Germany) (12.5 μL), primer F + R (1.5 μL + 1.5 μL), and sterile RNase free water (1.5 μL). Sequences of the tested primers, which were designed from tomato plants, are presented in Table 1. *β*-actine was used as a reference gene. The real time PCR program was carried out as follows: one cycle at 95 °C for 3 min, 45 cycles (95 °C for 15 s, 56 °C for 30 s, and 72 °C for 30 s). For each sample, three biological and three technical replicates were used. The comparative CT method (2^−ΔΔCT^) was used to analyze the relative expression level [29].

**Table 1:** Primer sequences of the studied genes [28]

Gene description	Abbrev.	Sequence (5′-3′)
Jasmonate and ethylene-responsive factor 3	*JERF3*-F	GCCATTTGCCTTCTCTGCTTC
	*JERF3*-R	GCAGCAGCATCCTTGTCTGA
Peroxidase	*POD*-F	CCTTGTTGGTGGGCACACAA
	*POD*-R	GGCCACCAGTGGAGTTGAAA
Chitinase class II	*CHI ΙΙ*-F	GCGTTGTGGTTCTGGATGACA
	*CHI ΙΙ*-R	CAGCGGCAGAATCAGCAACA
*β*-actin	*β*-actin-F	GTGGGCCGCTCTAGGCACCAA
	*β*-actin-R	CTCTTTGATGTCACGCACGATTTC

#### Disease assessment

Forty-five dpi, five maize plants from each treatment were carefully uprooted, washed under running tap water, and evaluated for incidence and severity of Fusarium root rot. Disease severity was evaluated by rating necrotic lesions on maize roots and hypocotyl on a 6-degrees scale described by McFadden et al. [30] and calculated as follows:

$$\mathrm{Disease}\ \mathrm{severity}\ \left(\%\right)=\frac{\sum ab}{AK}\times 100$$

Where, a = No. of diseased plants at the same degree, b = Degree of infection, A = total no. of evaluated plants and K = the highest degree of infection.

#### Plant growth evaluation

Forty-five dpi, five maize plants from each treatment were evaluated for shoot and root lengths, shoot and root dry weights, and number of leaves. Dry weights were recorded after the samples were dried in a drying oven at 80 °C until for 48 h.

#### Biochemical analyses

##### Preparation of crude extract

Forty-five dpi, maize roots (3 g) were ground in 5 ml phosphate buffer (100 mM, pH 7). The homogenate was centrifuged at 10,000 rpm for 15 min; the supernatant was then collected to serve as a crude extract in next analyses. For total phenol estimation, 1 g of maize roots was ground in 10 x volume of ethyl alcohol (85%), centrifuged at 5000 rpm for 15 min, and the supernatant was evaporated. The residue was then dissolved in 5 mL dist. Water. For each treatment, five replicates were used.

##### Estimation of total phenolic content and enzymes activities

Total phenolic content was estimated as described by Malik and Singh [31]. The reaction mixture contained 0.2 mL of the extract, dist. Water to 3 mL, and 0.5 mL Folin-Ciocalteu reagent. After 3 min, 2 mL Na_2_CO_3_ (20%) were added, and kept in boiling water for 1 min. The absorbance was measured at 650 nm. Activity of peroxidase (POD) enzyme was estimated according to Maxwell and Bateman [32]. The reaction mixture contained 12 mM K-phosphate buffer (pH 7.1), 4 mM pyrogallol, enzyme, and 1 mM H_2_O_2_. Change in absorbance was measured at 470 nm. Polyphenol oxidase (PPO) enzyme was determined according to Galeazzi et al. [33]. The reaction mixture contained 1 ml 0.2 M K-phosphate buffer (pH, 7.0), 0.5 ml crude extract and 0.5 ml catechol solution (100 mM). Change in absorbance was measured at 420 nm.

### Statistical analyses

The obtained data were analyzed using the software CoStat (version 6.4). Comparisons between the means were done using Tukey’s HSD test at *p* ≤ 0.05 based on one-way ANOVA.

## Results

### Molecular identification and phylogenetic analysis of endophytic fungus YRB2

**Fig. 1:**
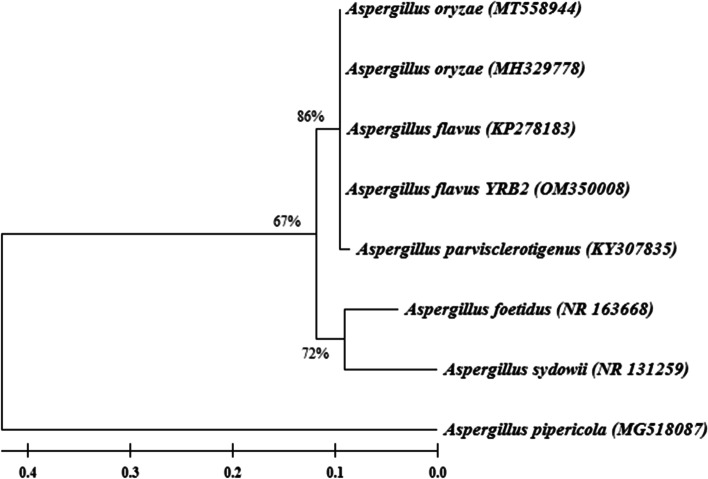
Phylogenetic tree showing the evolutionary relationship between the endophytic fungus YRB2 and the closest sequences from the GenBank database. Bootstrap values (%) are shown at the branches

Result from BLAST search analysis based on rDNA ITS region showed that the endophytic fungus YRB2 has 100% sequence similarity with *Aspergillus flavus*. The obtained nucleotide sequence (596 bp) was deposited in the GenBank database under accession number (OM350008). The phylogenetic analysis revealed that YRB2 was grouped into a distinct clade contained *A. flavus*, *A. oryzae,* and *A. parvisclerotigenus* with 85% bootstrap support, while *A. foetidus* and *A. sydowii* grouped in a separate clade with 70% bootstrap support. *Aspergillus pipericola* was used as an outgroup (Fig. 1).

### Antagonistic potential of *A. flavus* YRB2 against *F. solani* in vitro

**Fig. 2:**
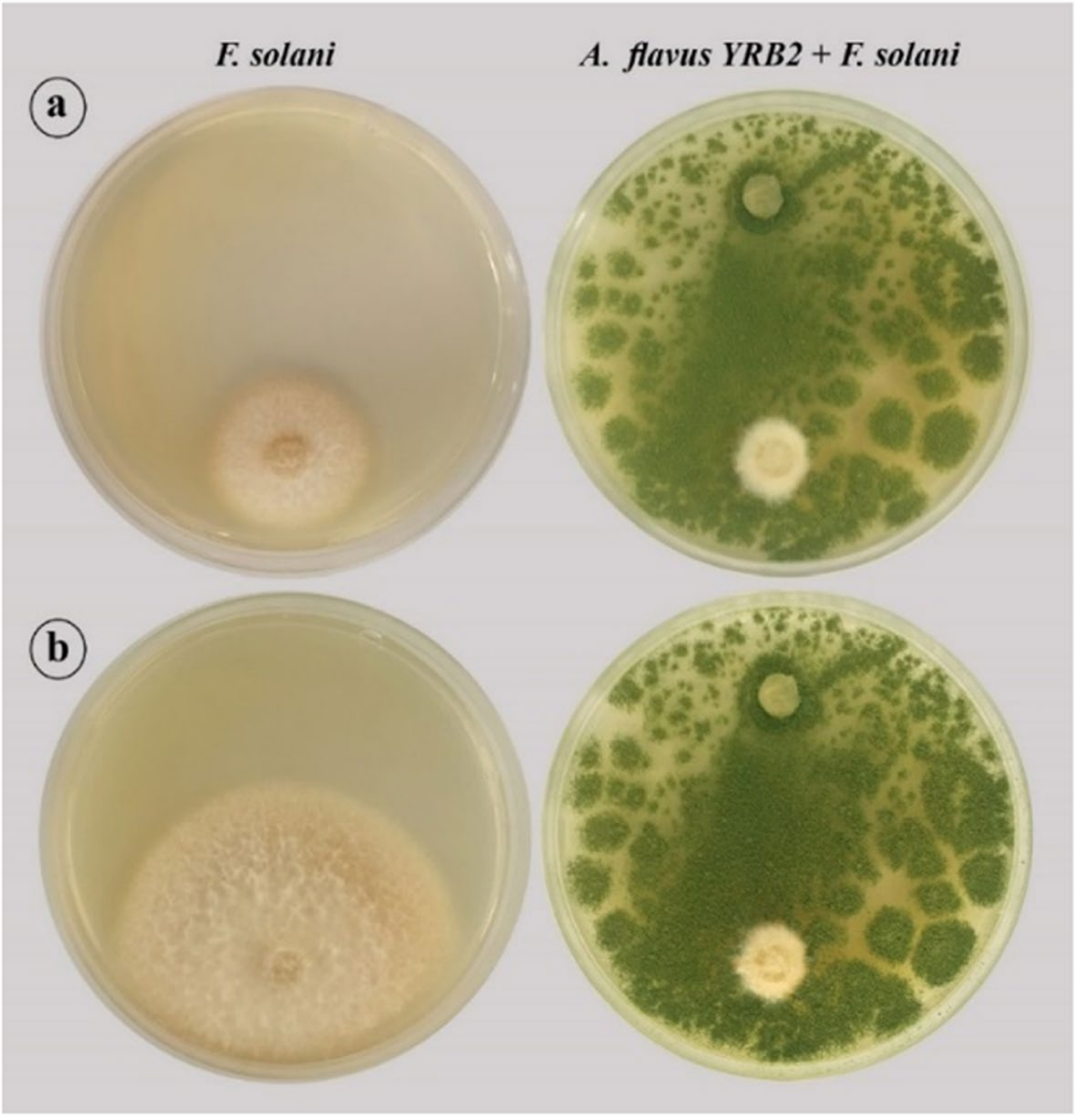
A dual culture test showing the antagonistic potential of *A. flavus* YRB2 against *F. solani* after 4 days (**a**) and 8 days (**b**)

Endophytic *A. flavus* YRB2 was screened for their antagonistic potential against *F. solani* in vitro (Fig. 2). Results obtained from the dual culture test revealed that growth rate of *A. flavus* YRB2 was higher than that of *F. solani*. A considerable inhibition in growth of *F. solani* was observed after 4 days incubation, compared to the control plate (Table 2). At day 8, growth of *F. solani* continued in the control plate recording 3.5 cm, while no further growth was achieved in the dual culture plate, recording 80% inhibition compared to the control plate. This result indicated the antagonistic potential of *A. flavus* YRB2 against *F. solani.*

**Table 2:** Antagonistic activity of endophytic *Aspergillus flavus* YRB2 against *Fusarium solani* in vitro^a^

Treatment	linear growth (cm) after		Growth Inhibition (%)
	4 days	8 days	
*F. solani*	1.5 ± 0.06^a^	3.5 ± 0.50^a^	0
*A. flavus* YRB2 + *F. solani*	0.7 ± 0.04^b^	0.7 ± 0.04^b^	80

^a^In each column, values followed by the same letter are not significantly different according to Tukey’s HSD test (*p* ≤ 0.05), each value represents the mean of five replicates ± SD

### Antifungal activity of extracellular metabolites of *A. flavus* YRB2

n-hexane and ethyl acetate extracts of *A. flavus* YRB2 culture filtrate were screened for their antifungal activity against *F. solani* in vitro*.* Data obtained revealed that n-hexane extract considerably suppressed the fungal growth of *F. solani*, recording 57.8% inhibition, compared with the untreated control (data not shown). On the contrary, the ethyl acetate extract of *A. flavus* YRB2 culture filtrate showed no inhibitory behavior against *F. solani.* Based on this result, the n-hexane extract was subjected for GC–MS analysis in order to identify its chemical constituents.

### GC-MS

**Fig. 3:**
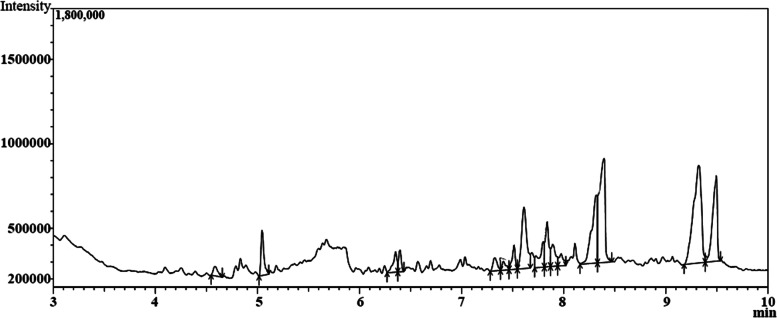
GC–MS chromatogram showing chemical constituents of n-hexane extract of *A. flavus* YRB2 culture filtrate. Arrows show peak limits

The GC-MS analysis for *A. flavus* YRB2 culture filtrate was performed to identify its produced secondary metabolites to whom the different bioactivities of the fungus can be attributed (Fig. 3). A total of 17 compounds was detected in varying proportions (Table 3). The major detected components included; palmitic acid (15.73%), α-linolenic acid (14.73%), stearic acid (12.86%), 3-methyl-5-propylnonane (10.26%), 8, 14-cedranoxide (9.26%), 2, 4-di-tert-butylphenol (6.82%), and 3, 5-di-tert-Butyl-ortho-benzoquinone (6.75%). Other constituents were detected in intermediate proportions such as eicosane (3.83%), diisobutyl phthalate (3.64%), heneicosane (3.45%), Palmitic acid, methyl ester (3.12%) and 2-hexyldecanol (3.05%). Whilst, the other detected constituents were of minor abundance.

**Table 3:** GC/MS analysis of n-hexane extract of *A. flavus* YRB2 culture filtrate

Peak #	Compound name	Retention time (min)	Molecular formula	Molecular weight (g/mol)
1	n-Octadecane	4.585	C_18_H_38_	254.49
2	2,4-Di-tert-butylphenol	5.045	C_14_H_22_O	206.32
3	2-hexyldecanol	6.355	C_16_H_34_O	242.44
4	Heneicosane	6.395	C_21_H_44_	296
5	1-chlorooctadecane	7.325	C_18_H_37_Cl	288.94
6	7,9-Di-tert-butyl-1-oxaspiro (4,5) deca-6,9-dien-2,8-dione	7.405	C_17_H_24_O_3_	276.37
7	diisobutyl phthalate	7.515	C_16_H_22_O_4_	278.34
8	8,14-Cedranoxide	7.610	C_15_H_24_O	220.35
9	3-Cyano-3-octyl-1,4-cyclohexadiene	7.695	C_15_H_23_N	217.35
10	Eicosane	7.795	C_20_H_42_	282
11	3,5-di-tert-Butyl-ortho-benzoquinone	7.840	C_14_H_20_O_2_	220.31
12	Palmitic acid, methyl ester	7.900	C_17_H_34_O_2_	270
13	Methyl 3-(3,5-di-tert-butyl-4-hydroxyphenyl) propionate	7.975	C_18_H_28_O_3_	292.41
14	3-Methyl-5-propylnonane	8.315	C_13_H_28_	184.36
15	n-Hexadecenoic acid (palmitic acid)	8.395	C_16_H_30_O_2_	254.41
16	9,12-(Z,Z)-Octadecadienoic acid (α-linolenic acid)	9.320	C_18_H_32_O_2_	280.45
17	Octadecanoic acid (stearic acid)	9.495	C_18_H_36_O_2_	284

### Aflatoxin production

**Fig. 4:**
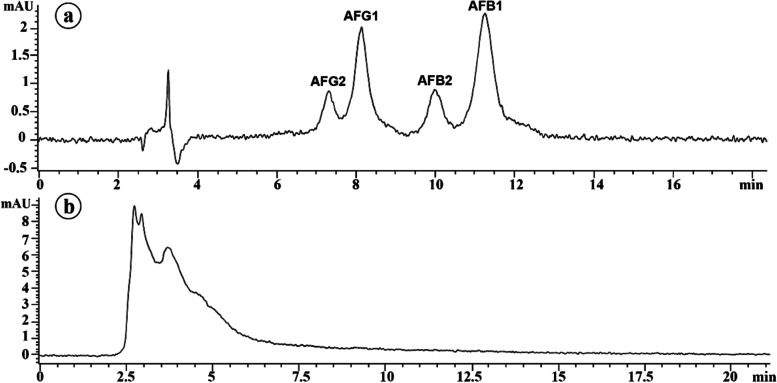
HPLC chromatogram of **a** aflatoxins mixture standard solution B1, B2, G1, and G2, and **b***A. flavus* YRB2 culture filtrate. AFB1, AFB2, AFG1, and AFG2 refer to aflatoxins B1, B2, G1, and G2, respectively

Production of aflatoxins B1, B2, G1, and G2 by *A. flavus* YRB2 was detected using HPLC system. The obtained HPLC chromatogram for the aflatoxins mixture standard solution (Fig. 4a) showed four distinct peaks for aflatoxins G2, G1, B2 and B1 at different retention times (7.313, 8.139, 9.980, and 11.254 min, respectively). In contrast, the HPLC chromatogram of *A. flavus* YRB2 culture filtrate did not show any corresponding peaks at these retention times (Fig. 4b). Absence of aflatoxins in the culture filtrate indicated that *A. flavus* YRB2 is a non-aflatoxigenic isolate.

### Expression profiling of the defense-related genes

**Fig. 5:**
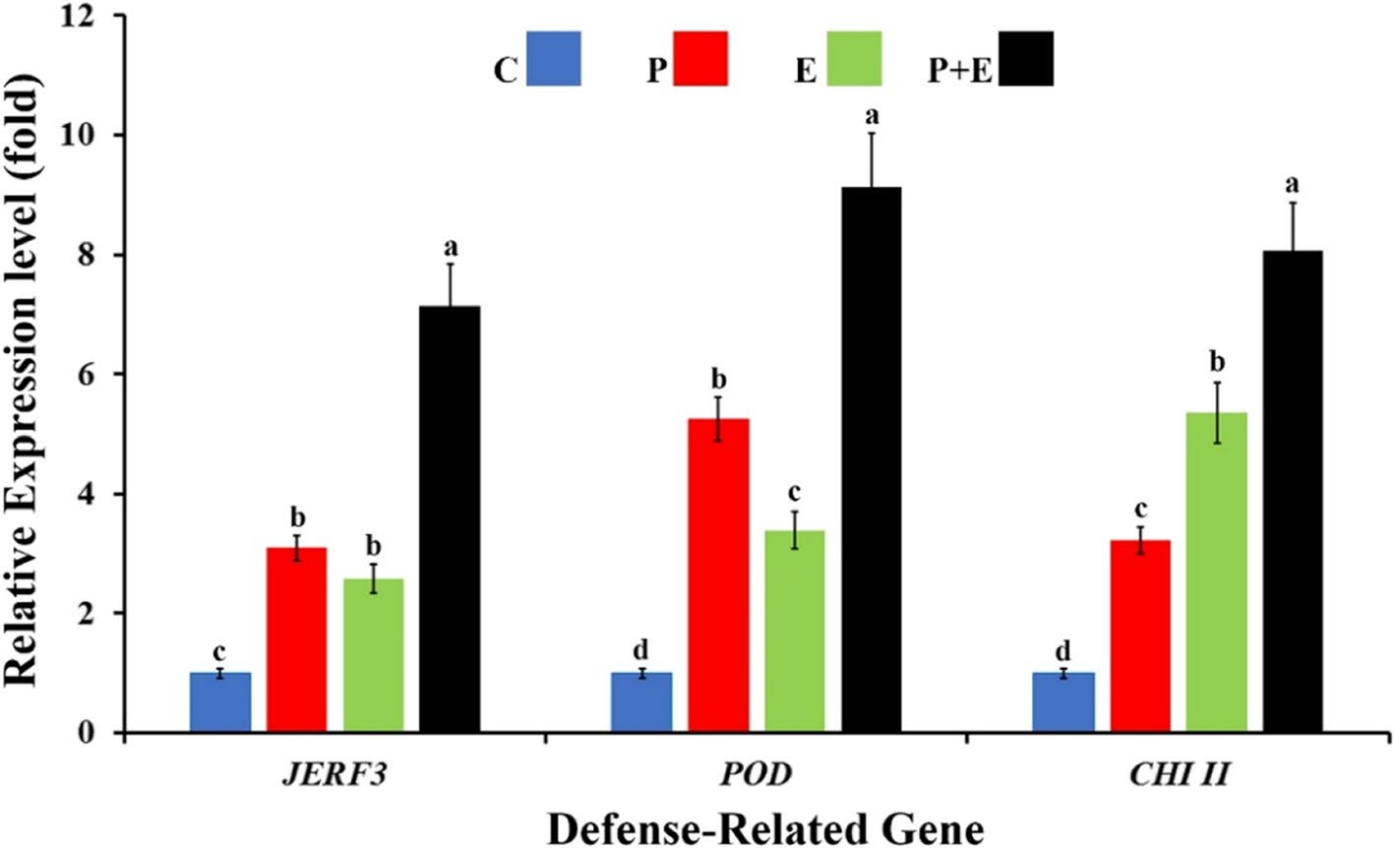
Gene expression profiles of jasmonate and ethylene-responsive factor 3 (*JERF3*), peroxidase (*POD*), and chitinase II (*CHI II*) in maize roots infected with Fusarium root rot and/or colonized with endophytic *A. flavus* YRB2, 3 days post-infection. Where, C: non-colonized with *A. flavus* YRB2 and uninfected with *F. solani*, P: non-colonized with *A. flavus* YRB2 and infected with *F. solani*, E: uninfected with *F. solani* and colonized with *A. flavus* YRB2, and P + E: infected with *F. solani* and colonized with *A. flavus* YRB2. For each gene, columns superscripted with the same letter are not significantly different according to Tukey’s HSD test at *p* ≤ 0.05. Each value is the mean of three biological replicates; each sample was analyzed in triplicate. Error bars represent standard errors

Expression profiles of the studied defense-related genes (*JERF3*, *POD*, and *CHI II*) of maize in response to infection with Fusarium root rot and/or colonization with endophytic *A. flavus* YRB2 at 3 dpi are illustrated in Fig. 5.

The obtained results showed that infection with Fusarium root rot or colonization with endophytic *A. flavus* YRB2 overexpressed the responsive factor *JERF3*, compared with the untreated control. No significant difference was detected between both treatments. However, the highest expression level was recorded for maize plants infected with Fusarium root rot and colonized with *A. flavus* YRB2 (7.2-fold), when compared with the control plants. For *POD*, data obtained indicated that all applied treatments upregulated the transcriptional expression of *POD*, at varying extents, compared with the untreated plants. In this concern, the expression profiles obtained by qRT-PCR showed that infection of maize plants with *F. solani* was more inducer than colonization with *A. flavus* YRB2. The highest expression level was observed for the infected maize plants, which colonized with *A. flavus* YRB2 (9.1-fold). Regarding *CHI II,* the obtained results revealed that all tested treatments triggered the gene expression at varying extents, compared with the untreated maize plants. However, colonization with endophytic *A. flavus* YRB2 had more triggering effect than infection with Fusarium root rot. While the highest expression level was recorded for maize plants infected with Fusarium root rot and colonized with *A. flavus* YRB2 (8-fold).

### Disease assessment

Maize plants infected with *F. solani* were evaluated for disease incidence and severity in response to colonization with endophytic *A. flavus* YRB2 at 45 dpi (Table 4). Maize plants only infected with *F. solani* showed typical symptoms of Fusarium root rot, recording 100% incidence and 78.3% severity. No disease incidence or severity were observed for maize plants not infected with *F. solani* or that colonized only with *A. flavus* YRB2. Results obtained from the greenhouse experiment showed that colonization of the infected maize plants with *A. flavus* YRB2 reduced the disease severity and incidence recording 73.4% inhibition.

**Table 4:** Disease severity of maize plants infected with Fusarium root rot in response to colonization with endophytic *Aspergillus flavus* YRB2 at 45 days post infection*

Treatment	Disease incidence (%)	Disease severity (%)	Reduction (%)
C	0.0^c^	0.0^c^	0.0^b^
P	100.0 ± 0.2^a^	78.3 ± 2.4^a^	0.0^b^
E	0.0^c^	0.0^c^	0.0^b^
P + E	67.6 ± 1.5^b^	20.8 ± 1.7^b^	73.4 ± 3.1^a^

*In each column, values followed by the same letter are not significantly different according to Tukey’s HSD test (*p* ≤ 0.05), each value represents the mean of five replicates ± SD. Where, C: non-colonized with *A. flavus* YRB2 and uninfected with *F. solani*, P: non-colonized with *A. flavus* YRB2 and infected with *F. solani*, E: uninfected with *F. solani* and colonized with *A. flavus* YRB2, and P + E: infected with *F. solani* and colonized with *A. flavus* YRB2

### Plant growth evaluation

Mean growth parameters of maize plants infected with *F. solani* in response to colonization with endophytic *A. flavus* YRB2 at 45 dpi are presented in Table 5. Compared with the non-colonized-uninfected maize plants, data from the greenhouse experiment revealed the growth promoting effect of colonization with *A. flavus* YRB2 on shoot and root lengths, as well as shoot and root dry weights, but not leaves number, recording the highest values in this concern. In contrast, infection with Fusarium root rot significantly reduced the evaluated growth parameters, except leaves number, when compared with the untreated control plants. Furthermore, colonization of the infected maize plants with *A. flavus* YRB2 significantly enhanced their growth parameters, except leaves number, compared with the infected plants not colonized with *A. flavus* YRB2.

**Table 5:** Growth parameters of maize plants infected with Fusarium root rot in response to colonization with endophytic *Aspergillus flavus* YRB2 at 45 days post infection*

Treatment	Shoots length (cm)	Root length (cm)	Shoots dry weight (g)	Root dry weight (g)	Number of leaves
C	49.3 ± 1.5^b^	16.7 ± 0.6^b^	0.94 ± 0.03^ab^	0.52 ± 0.02^b^	4.0 ± 0.2^a^
P	38.1 ± 1.2^c^	13.1 ± 0.4^c^	0.76 ± 0.05^c^	0.39 ± 0.03^c^	4.0 ± 0.1^a^
E	55.7 ± 1.6^a^	18.7 ± 0.5^a^	1.02 ± 0.04^a^	0.60 ± 0.02^a^	5.0 ± 0.2^a^
P + E	46.7 ± 1.5^b^	15.8 ± 0.7^b^	0.90 ± 0.01^b^	0.49 ± 0.05^b^	4.0 ± 0.1^a^

*In each column, values followed by the same letter are not significantly different according to Tukey’s HSD test (*p* ≤ 0.05), each value represents the mean of five replicates ± SD. Where, C: non-colonized with *A. flavus* YRB2 and uninfected with *F. solani*, P: non-colonized with *A. flavus* YRB2 and infected with *F. solani*, E: uninfected with *F. solani* and colonized with *A. flavus* YRB2, and P + E: infected with *F. solani* and colonized with *A. flavus* YRB2

### Total phenolic content and enzymes activities

Mean total phenolic content and activities of the antioxidant enzymes POD and PPO in maize roots in response to infection with *F. solani* and/or colonization with endophytic *A. flavus* YRB2 at 45 dpi are presented in Table 6. Results from the biochemical analyses showed that either infection with *F. solani* or colonization with endophytic *A. flavus* YRB2 significantly led to an increment in the total phenolic content and induced the enzyme activity of POD and PPO, compared with the uninfected-non-colonized maize plants. In this regard, the triggering effect of infection with *F. solani* was higher than that due to colonization with *A. flavus* YRB2. However, the highest values were recorded for maize plants infected with *F. solani* and colonized with *A. flavus* YRB2, compared with the uninfected-non-colonized control plants.

**Table 6:** Total phenolic content and activities of peroxidase and polyphenol oxidase enzymes of maize roots infected with Fusarium root rot in response to colonization with endophytic *Aspergillus flavus* YRB2 at 45 days post infection*

Treatment	Total phenols (mg.g^− 1^ fresh wt)	Peroxidase (∆A_470_ min^− 1^ g^− 1^ fresh wt)	Polyphenol oxidase (∆A_420_ min^− 1^ g^− 1^ fresh wt)
C	115.0 ± 4.5^d^	1.053 ± 0.1^d^	1.522 ± 0.1^d^
P	272.3 ± 3.2^b^	2.941 ± 0.2^b^	2.033 ± 0.2^b^
E	215.4 ± 3.6^c^	2.520 ± 0.2^c^	1.791 ± 0.1^c^
P + E	306.1 ± 3.5^a^	3.245 ± 0.1^a^	2.787 ± 0.2^a^

*In each column, values followed by the same letter are not significantly different according to Tukey’s HSD test (*p* ≤ 0.05), each value represents the mean of five replicates ± SD. Where, C: non-colonized with *A. flavus* YRB2 and uninfected with *F. solani*, P: non-colonized with *A. flavus* YRB2 and infected with *F. solani*, E: uninfected with *F. solani* and colonized with *A. flavus* YRB2, and P + E: infected with *F. solani* and colonized with *A. flavus* YRB2

## Discussion

Endophytic microorganisms represent a promising source of natural antimicrobial substances, biofertilizers, and growth hormones, which can be used as sustainable approaches to reduce the use of agrochemicals [34, 35]. In this study, antagonistic activity of endophytic *A. flavus* YRB2 against *F. solani* in vitro and its biocontrol activity against Fusarium root rot of maize under greenhouse conditions were studied. Results obtained revealed that *A. flavus* YRB2 exhibited great antagonistic potential against *F. solani.* This result is in agreement with that obtained by Campos and Jacob [36] who reported potential antagonistic activity for three endophytic *Aspergillus* spp., isolated from Mexican mint leaves, against *F. verticillioides*, the causal of maize stalk rot. Endophytic microorganisms represent a vital source of a plethora of bioactive compounds [16]. Data from the GC-MS analysis indicated that *A. flavus* YRB2 produced a diverse set of secondary metabolites with antifungal background that may explain its great antagonistic behavior against *F. solani*. Among the identified secondary metabolites, three fatty acids, namely palmitic acid, α-linolenic acid, and stearic acid constituted 43.3% of the produced metabolites. Antifungal activity of these fatty acids has been reported in many researches [37, 38]. Different modes of action have been discussed in this concern including disruption of the cell membrane permeability leading to electrolyte leakage and cell death, and inhibition of protein synthesis and fatty acids metabolism [39]. The volatile phenolic compound 2, 4-di-tert-butylphenol is another antifungal metabolite which produced by *A. flavus* YRB2. Varsha et al. [40] reported a potent antifungal activity for this compound against different pathogenic fungi such as *A. niger*, *F. oxysporum* and *Penicillium chrysogenum*. Moreover, a high free radical scavenging activity, and a biocontrol activity against these fungi on wheat grains were also reported [41, 42]. The proposed antifungal mechanism of this phenolic compound is the suppression of spore germination, and inhibition of cell mitotic division [43]. In addition, diisobutyl phthalate and heneicosane are another antifungal metabolites which produced by *A. flavus* YRB2 [44, 45]. All of these compounds synergistically contributed to the aggressive nature of *A. flavus* YRB2. HPLC analysis showed absence of aflatoxins B1, B2, G1, and G2 in the culture filtrate of *A. flavus* YRB2, confirming that it is non-aflatoxigenic. This result is in agreement with that obtained by Balbinot et al. [46] who isolated the non-aflatoxigenic, endophytic *A. flavus* CL7 from the wild shrub *Chromolaena laevigata*. Isolation of non-aflatoxigenic strains of *A. flavus* has been widely reported in the last years, where used as a promising strategy for biocontrol of aflatoxigenic strains, and prevention/reduction of aflatoxin contamination under field conditions [47]. Use of non-aflatoxigenic strains of *A. flavus* as competitor fungi was found to reduce/displace aflatoxigenic strains populations, and considerably reduce their aflatoxin production, which is called the displacement strategy for biocontrol [48]. It was suggested that absence of aflatoxigenicity in *A. flavus* might be an adaptation result for the carbon-rich condition, which affects genetic stability of the aflatoxin biosynthesis pathway gene cluster [49].

Results obtained from the greenhouse experiment showed the biocontrol activity of *A. flavus* YRB2 in reducing the disease severity and incidence of maize plants. This result is in accordance with the findings obtained by Karunasinghe et al. [50] who reported a 70% reduction in damping off of cucumber, caused by *Pythium aphanidermatum*, in response to application of the endophytic *A. insulicola* A435 or *A. luchuensis* A116. Many recent researches showed that different endophytic fungi successfully act as biocontrol agents against various phytopathogens by eliciting the plant resistance [51]. Taking in consideration the selectivity between the plant and the endophyte, different co-evolutionary adaptations are expected between them via intense chemotactic signaling [52]. In this study, overexpression of the defense-related genes *JERF3*, *POD*, and *CHI II* in maize plants due to colonization with *A. flavus* YRB2 was observed. *JERF3* regulates many defense-related genes via jasmonate and ethylene signaling pathways triggering the plant responses against different biotic and abiotic stresses [20]. *CHI* is a defensive gene, which catalyzes degradation of chitin, the main structural unit of the cell wall in fungi [22]. Overexpression of the antioxidant stress marker gene (*POD*) is one of the reported defense responses scavenging the ROS resulted in the plant tissue due to different stresses [23]. This defense response was supported by the increment in the activities of POD and PPO enzymes reported in this study. Upregulation of these defense-related genes contributed in minimizing the disease severity and incidence observed in maize plants, in response to colonization with *A. flavus* YRB2. Moreover, accumulation of fungitoxic phenolic compounds was another observed mechanism in maize plants, which may play a role in restriction of growth of *F. solani* and reducing its transfer from cell to cell. This result is in agreement with that obtained by Rashad et al. [53] who reported an increment in the total phenolic compounds in garlic plants colonized with endophytic *Bacillus amyloliquefaciens* GGA and mycorrhizal fungi against infection with white rot. In general, colonization of a plant with endophytic fungi triggers production of defensive secondary metabolites by the endophyte or the host, modifies its morphology, and/or induces its resistance responses via activation of different signaling pathways defending itself against the invading pathogens [51]. The proposed defensive mechanisms include cell wall lignification, accumulation of fungitoxic secondary metabolites, stimulation the host to produce phytoalexins and PR-proteins, and/or overexpression of various defense-related genes [19].

Our results indicated the growth promoting effect of colonization of maize plants with *A. flavus* YRB2. This result is in accordance with the findings obtained by Hamayun et al. [54] who reported a growth promoting effect for *A. flavus* EuR-6, isolated from *Euphorbia indica*, on soybean and sunflower seedlings even under heat stress conditions. Moreover, this fungal endophyte was found to produce indole acetic acid (IAA) in a considerable amount. Use of beneficial endophytic microorganisms as biofertilizers for improving plant growth and production is crucial for sustainable agriculture and food safety [35]. Different mechanisms are discussed in this concern including production of growth promoting metabolites such as phytohormones, mobilization of unavailable nutrients, and/or mitigation biotic and abiotic stresses [16]. The phytohormone IAA induces cell division and differentiation, triggers lateral growth, enhances the growth rate of tissue development, promotes pigments production, improves plant metabolism, and regulates defensive responses to alleviate stress conditions [55]. Therefore, IAA production by endophytes is recognized as the most vital bioactive molecule in growth promotion, and plant-endophyte interactions. In addition, gibberellins have been reported to be produced by the endophytic fungi. Waqas et al. [56] reported production of the gibberellins GA1, GA3, GA4, and GA7) in varying quantities by *Phoma glomerata* LWL2 and *Penicillium* sp. LWL3, isolated from cucumber plants. Inorganic phosphate solubilization is another plant growth promoting mechanism of endophytic fungi through production of phosphate-solubilizing enzymes such as phosphatases and phytases, and/or organic acids. In this regard, Adhikari and Pandey [57] reported production of phosphatase and phytase enzymes, as well as malic, succinic, oxalic, lactic and citric acids by five endophytic fungal species of the genera *Aspergillus* and *Penicillium*, isolated from *Taxus wallichiana*. Mineralization of organophosphorus compounds by fungal endophytes has a vital role in enhancing the plant phosphorus uptake, and the soil fertility. Production of siderophores, which chelate iron making it available to the plant, is one of the growth-promoting mechanisms utilized by endophytic fungi [58]. *Aspergillus* spp. have been widely reported as producers of siderophores, acting as a model organism for studying metabolism of these bioactive metabolites [59]. Iron acquisition by endophytic fungi has many beneficial roles in plant growth, enzymatic metabolism, antioxidant responses, and symbiotic interactions [60].

## Conclusions

Results obtained in this study showed a potent antagonistic behavior for endophytic *A. flavus* YRB2 against *F. solani* in vitro. A set of antifungal secondary metabolites were found to be produced by this endophyte. In contrast, no aflatoxins (B1, B2, G1, and G2) were detected to be produced by this endophyte, indicating that it is a non-aflatoxigenic strain. Under greenhouse conditions, *A. flavus* YRB2 exhibited a potential biocontrol activity against Fusarium root rot of maize. Overexpression of the defense-related genes (*JERF3*, *CHI II*, and *POD*) was reported, indicating the inducing effect on the plant immunity. In addition, an increment in the antioxidant enzymes POD and PPO, and the total phenolic content was also observed in response to this treatment. Moreover, a growth-promoting effect was also observed for *A. flavus* YRB2 on maize plants. Based on the obtained data, we can conclude that *A. flavus* YRB2 may represent a promising biocontrol and growth-promoting agent in maize plants against Fusarium root rot. Nevertheless, field evaluation is highly requested before the use recommendation.

## Data Availability

rDNA ITS sequencing raw data of *A. flavus* YRB2 was deposited to the GenBank database under accession number (OM350008).
